# Evidence for Reductive Genome Evolution and Lateral Acquisition of Virulence Functions in Two *Corynebacterium pseudotuberculosis* Strains

**DOI:** 10.1371/journal.pone.0018551

**Published:** 2011-04-18

**Authors:** Jerônimo C. Ruiz, Vívian D'Afonseca, Artur Silva, Amjad Ali, Anne C. Pinto, Anderson R. Santos, Aryanne A. M. C. Rocha, Débora O. Lopes, Fernanda A. Dorella, Luis G. C. Pacheco, Marcília P. Costa, Meritxell Z. Turk, Núbia Seyffert, Pablo M. R. O. Moraes, Siomar C. Soares, Sintia S. Almeida, Thiago L. P. Castro, Vinicius A. C. Abreu, Eva Trost, Jan Baumbach, Andreas Tauch, Maria Paula C. Schneider, John McCulloch, Louise T. Cerdeira, Rommel T. J. Ramos, Adhemar Zerlotini, Anderson Dominitini, Daniela M. Resende, Elisângela M. Coser, Luciana M. Oliveira, André L. Pedrosa, Carlos U. Vieira, Cláudia T. Guimarães, Daniela C. Bartholomeu, Diana M. Oliveira, Fabrício R. Santos, Élida Mara Rabelo, Francisco P. Lobo, Glória R. Franco, Ana Flávia Costa, Ieso M. Castro, Sílvia Regina Costa Dias, Jesus A. Ferro, José Miguel Ortega, Luciano V. Paiva, Luiz R. Goulart, Juliana Franco Almeida, Maria Inês T. Ferro, Newton P. Carneiro, Paula R. K. Falcão, Priscila Grynberg, Santuza M. R. Teixeira, Sérgio Brommonschenkel, Sérgio C. Oliveira, Roberto Meyer, Robert J. Moore, Anderson Miyoshi, Guilherme C. Oliveira, Vasco Azevedo

**Affiliations:** 1 Research Center René Rachou, Oswaldo Cruz Foundation, Belo Horizonte, Minas Gerais, Brazil; 2 Department of General Biology, Federal University of Minas Gerais, Belo Horizonte, Minas Gerais, Brazil; 3 Department of Genetics, Federal University of Pará, Belém, Pará, Brazil; 4 Health Sciences Center, Federal University of São João Del Rei, Divinópilis, Minas Gerais, Brazil; 5 Department of Veterinary Medicine, State University of Ceará, Fortaleza, Ceará, Brazil; 6 Department of Genetics, University of Bielefeld, CeBiTech, Bielefeld, Nordrhein-Westfale, Germany; 7 Department of Computer Science, Max-Planck-Institut für Informatik, Saarbrücken, Saarlan, Germany; 8 Department of Pharmaceutical Sciences, Federal University of Ouro Preto, Ouro Preto, Minas Gerais, Brazil; 9 Department of Phisics, Federal University of Ouro Preto, Ouro Preto, Minas Gerais, Brazil; 10 Department of Biological Sciences, Federal University of Triangulo Mineiro, Uberaba, Minas Gerais, Brazil; 11 Department of Genetics and Biochemistry, Federal University of Uberlândia, Uberlândia, Minas Gerais, Brazil; 12 Brazilian Agricultural Research Corporation (EMBRAPA), Sete Lagoas, Minas Gerais, Brazil; 13 Department of Biochemistry and Immunology, Federal University of Minas Gerais, Belo Horizonte, Minas Gerais, Brazil; 14 Department of Parasitology, Federal University of Minas Gerais, Belo Horizonte, Minas Gerais, Brazil; 15 Department of Pharmacy, Federal University of Ouro Preto, Ouro Preto, Minas Gerais, Brazil; 16 Department of Technology, State University of São Paulo, Jaboticabal, São Paulo, Brazil; 17 Department of Chemistry, Federal University of Lavras, Lavras, Minas Gerais, Brazil; 18 Brazilian Agricultural Research Corporation (EMBRAPA), Campinas, São Paulo, Brazil; 19 Department of Plant Pathology, Federal University of Viçosa, Viçosa, Minas Gerais, Brazil; 20 Department of Biointeraction Sciences, Federal University of Bahia, Salvador, Bahia, Brazil; 21 CSIRO Livestock Industries, Australia; 22 Center of Excellence in Bioinformatics, National Institute of Science and Technology, Research Center René Rachou, Oswaldo Cruz Foundation, Belo Horizonte, Minas Gerais, Brazil; St. Petersburg Pasteur Institute, Russian Federation

## Abstract

**Background:**

*Corynebacterium pseudotuberculosi*s, a Gram-positive, facultative intracellular pathogen, is the etiologic agent of the disease known as caseous lymphadenitis (CL). CL mainly affects small ruminants, such as goats and sheep; it also causes infections in humans, though rarely. This species is distributed worldwide, but it has the most serious economic impact in Oceania, Africa and South America. Although *C. pseudotuberculosis* causes major health and productivity problems for livestock, little is known about the molecular basis of its pathogenicity.

**Methodology and Findings:**

We characterized two *C. pseudotuberculosis* genomes (Cp1002, isolated from goats; and CpC231, isolated from sheep). Analysis of the predicted genomes showed high similarity in genomic architecture, gene content and genetic order. When *C. pseudotuberculosis* was compared with other *Corynebacterium* species, it became evident that this pathogenic species has lost numerous genes, resulting in one of the smallest genomes in the genus. Other differences that could be part of the adaptation to pathogenicity include a lower GC content, of about 52%, and a reduced gene repertoire. The *C. pseudotuberculosis* genome also includes seven putative pathogenicity islands, which contain several classical virulence factors, including genes for fimbrial subunits, adhesion factors, iron uptake and secreted toxins. Additionally, all of the virulence factors in the islands have characteristics that indicate horizontal transfer.

**Conclusions:**

These particular genome characteristics of *C. pseudotuberculosis*, as well as its acquired virulence factors in pathogenicity islands, provide evidence of its lifestyle and of the pathogenicity pathways used by this pathogen in the infection process. All genomes cited in this study are available in the NCBI Genbank database (http://www.ncbi.nlm.nih.gov/genbank/) under accession numbers CP001809 and CP001829.

## Introduction

*Corynebacterium pseudotuberculosis* is a facultative intracellular pathogen that mainly infects sheep and goats, causing the disease called caseous lymphadenitis (CL). This bacterium can also cause ulcerative lymphangitis in equines; superficial abscesses in bovines, pigs, deer and laboratory animals; arthritis and bursitis in ovines; pectoral abscesses in equines and, more rarely, in camels, caprines and deer [1]-[3]. In both disease manifestations, its main characteristic is abscessing of the lymph nodes [4]. Rare cases of human infection have also been reported [5], [6].

Despite the broad spectrum of hosts, the high incidence of CL reported from various countries, including Australia, New Zealand, South Africa, the United States of America, Canada and Brazil, mainly refers to small ruminants [7]-[11]. According to the World Animal Health Organization, among 201 countries that reported their sanitary situations, 64 declared the presence of animals with CL within their borders (OIE, 2009). The highest prevalence of CL has been reported in Brazil [12]. Pinheiro and colleagues (2000) reported 66.9% of animals with clinical signs of CL in the state of Ceará. In Minas Gerais state, a prevalence of 75.8% was reported for sheep [13] and 78.9% for goats [14]. In Australia, 61% of sheep flocks showed signs of infection [15]. In the USA, the prevalence ranges up to 43% [16]. Similar levels have been reported from the Canadian province of Quebec, with a prevalence of 21 to 36% [10]. In the United Kingdom, 45% of the producers that were polled reported abscesses in their sheep [9].

The high prevalence of CL in sheep and goats has made studies on ways to detect *C. pseudotuberculosis* in these hosts increasingly important; an efficient means to accomplish this would be a valuable tool for the control of this disease. Currently, there is no sufficiently sensitive and specific diagnostic test for subclinical CL. Diagnosis is currently achieved only by routine bacterial culture of purulent material collected from animals that have external abscesses, with subsequent biochemical identification of the isolates [17]. A few vaccines against CL are currently available, although they have not been licensed for use in many countries. Not all vaccines that have been developed for sheep are effective in goats. It is usually necessary to adjust vaccination programs to each animal host species [18].

Considering the current unfortunate status of CL prevalence in the world, especially in Brazil and Australia, there is a pressing need for more efficient alternatives for disease control that not only cure sick animals but also minimize or even prevent the onset of disease in herds. One of the major efforts to eradicate this disease involves the identification of genes that are related to the *C. pseudotuberculosis* pathogenicity and lifestyle. As an intracellular facultative pathogen, *C. pseudotuberculosis* exhibits several characteristics in its genome, such as gene loss, low GC content and a reduced genome [19] that differ from those of non-pathogenic *Corynebacterium* species. The finding of seven putative pathogenicity islands containing classical virulence elements, including genes for iron uptake, fimbrial subunits, insertional elements and secreted toxins [20], probably mostly acquired through horizontal transfer, contributes to our understanding of how this species causes disease. Comprehensive knowledge of an organism's genome facilitates an exhaustive search for candidates for virulence genes, vaccine and antimicrobial targets, and components that could be used in diagnostic procedures.

The information retrieved from a single genome is insufficient to provide an understanding of all *C. pseudotuberculosis* strains. Comparative genomics can shed light on the molecular attributes of a strain that affect its virulence, host specificity, dissemination potential and resistance to antimicrobial agents [21], [22]. Furthermore, comparison of entire genome sequences of strains belonging to the same species, but from different geographic, epidemiological, chronological and clinical backgrounds, as well as affecting different hosts, would be useful for determining the molecular basis of these differences. As part of an effort to provide means to control CL, we examined the genomes of two strains of *C. pseudotuberculosis* isolated from sheep and goats, respectively, and compared them to each other and to the genomes of two other strains already available in a public database [6], [23].

## Results

### *Corynebacterium pseudotuberculosis* genome

**Figure 1:**
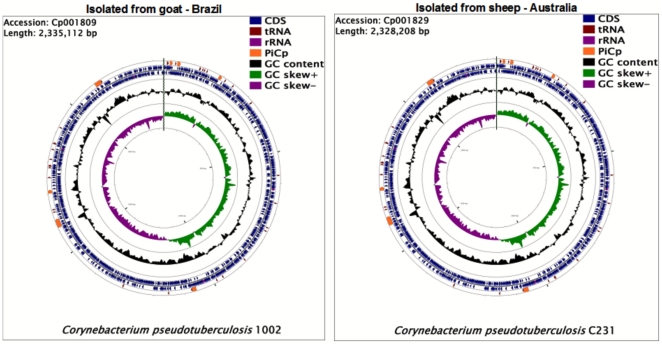
The whole genome of *Corynebacterium pseudotuberculosis.* Cp1002 strain isolated from a goat in Brazil and CpC231 strain isolated from sheep in Australia. Highlighted in yellow are the pathogenicity islands (PiCps) of *C. pseudotubeculosis* and its location in the genomes.

Overviews of the *C. pseudotuberculosis* genomes can be seen in Figure 1. The genomes are available in the NCBI GenBank database under accession numbers Cp1002:CP001809 and CpC231:CP001829.

The two strains are very similar, with an amino acid similarity of at least 95% between their predicted proteins. In their genomic composition, the isolates were found to have the same mean i) GC content, ii) gene length, iii) operon composition and iv) gene density. However, some significant differences were observed in: i) genome size, ii) number of pseudogenes and iii) lineage-specific genes (Table 1).

**Table 1:** General features of the genomes of two *Corynebacterium pseudotuberculosis* strains.

Genome feature	Cp1002	CpC231
Genome size (bp)	2,335,112	2,328,208
Gene number	2111	2103
Operon predicted number	474	468
Pseudogene number	53	50
tRNA number	48	48
rRNA operon	4	4
Gene mean length (bp)	964	968
Gene density (%)	0.88	0.88
Coding percentage	84.9	85.4
GC content (gene) (%)	52.88	52.86
GC content (genome) (%)	52.19	52.19
Lineage-specific genes	52	49

### Gene order in *C. pseudotuberculosis*

To determine whether synteny was maintained between the two *C. pseudotuberculosis* strains, we made a comparative analysis of global gene order. As expected, the two *C. pseudotuberculosis* strains showed high synteny conservation; approximately 97% of their genes were found to be conserved in the comparison between the two strains. Previous studies provide evidence of a high degree of conservation of gene order in four *Corynebacterium* genomes, *C. diphtheriae*, *C. glutamicum*, *C. efficiens* and *C. jeikeium*, showing only 10 gene-order breakpoints; rearrangement events during evolution in this species appear to be rare [24], [25]. We checked the validity of this conclusion by making a comparative analysis of the genomes of the two *C. pseudotuberculosis* strains against *C. diphtheriae*, the *Corynebacterium* species that is most closely related to *C. pseudotuberculosis* [26], [27].

Both *C. pseudotuberculosis* genomes showed a high degree of conservation in gene position, when compared to the *C. diphtheriae* genome, with few rearrangement points. This finding supports the hypothesis of a high degree of synteny conservation in this genus [25].

### Pathogenicity islands (PAIs)

Pathogenicity islands in bacterial genomes can be characterized by looking for characteristics linked to horizontal gene transfer, such as differences in codon usage, G+C content, dinucleotide frequency, insertion sequences, and tRNA flanking regions, together with transposase coding genes, which are involved in incorporation of DNA by transformation, conjugation or bacteriophage infection [28].

Pathogenicity islands had not been reported for *C. pseudotuberculosis*; to date; we used a multi-pronged approach called PIPS (submitted article) to identify the putative PAIs of *C. pseudotuberculosis*. Seven regions with most or all of the characteristics of horizontally-acquired DNA were found in both strains, Cp1002 and CpC231: i) base composition and/or codon usage deviations, ii) tRNA flanking, and iii) transposase genes. These regions were not found in a non-pathogenic species belonging to the same genus, *C. glutamicum*, and were classified as putative pathogenicity islands in *C. pseudotuberculosis* (PiCp). PiCps encode for proteins involved in the ABC transport system, for glycosil transferase, a two-component system, the *fag* operon and phospholipase D Table 2 provides a list of some genes found in the PAIs, with their respective functions.

**Table 2:** Genes and proteins present in pathogenicity islands of the *Corynebacterium pseudotuberculosis* strain genomes.

PAI	Cp1002	CpC231	Protein
	tnp7109-9	tnp7109-9	Transposase for insertion sequence
	pld	pld	Phospholipase D precursor (PLD)
PiCp 1	fag C	fag C	ATP binding cytoplasmic membrane protein - FagC
	fag B	fag B	Iron-enterobactin transporter - FagB
	fag A	fag A	Integral membrane protein - FagA
	fag D	fag D	Iron siderophore binding protein - FagD
	mgtE	mgtE	Mg2+ transporter mgtE
	malL	malL	Oligo-1,6-glucosidase
PiCp 2	tetA	tetA	Putative tetracycline-efflux transporter
	cskE	cskE	Anti-sigma factor
	sigK	sigK	ECF family sigma factor K
	dipZ	dipZ	Integral membrane C-type cytochrome biogenesis protein DipZ
	potG	potG	Putrescine ABC transport system
	afuB	afuB	Putative transport system permease (iron)
PiCp 3	afuA	afuA	Iron (Fe3+) ABC superfamily ATP binding cassette transporter, binding protein
	glpT	glpT	Glycerol-3-phosphate transporter
	phoB	phoB	Two-component regulatory protein
	lcoS	lcoS	Two-component sensor protein, sensor histidine kinase
	ciuA	ciuA	Putative iron transport system binding (secreted) protein
	ciuB	ciuB	Putative iron transport system membrane protein
PiCp 4	ciuC	ciuC	Putative iron transport system membrane protein
	ciuD	ciuD	Putative iron ABC transport system
	ciuE	ciuE	Putative siderophore biosynthesis related protein
	σ70	σ70	Putative RNA polymerase sigma factor 70
	Pseudogene	Pseudogene	Putative chromosome segregation ATPase
PiCp 5	hsdR	hsdR	Putative type III restriction-modification system
	pfoS	pfoS	PfoR superfamily protein
	htaC	htaC	HtaA family protein
	guaB3	guaB3	Inosine 5-monophosphate dehydrogenase
PiCp6	pipA1	pipB	Proline iminopeptidase
	mfsD1	mfsD1	Major facilitator superfamily domain-containing protein 1
	dcd	dcd	Deoxycytidine triphosphate deaminase
	udg	udg	UDP-glucose 6-dehydrogenase
	lysS1	lysS1	Lysyl-tRNA synthetase
	alaT	alaT	Aminotransferase AlaT
	ureA	ureA	Urease gamma subunit
	ureB	ureB	Urease beta subunit
	ureC	ureC	Putative urease subunit alpha
PiCp 7	ureE	ureE	Urease accessory protein
	ureF	ureF	Urease accessory protein
	ureG	ureG	Urease accessory protein
	ureD	ureD	Urease accessory protein
	fepC2	fepC2	ABC superfamily ATP binding cassette transporter
	fecD	fecD1	Iron(III) dicitrate transport system permease fecD
	phuC	phuC	Iron(III) dicitrate transport permease-like protein yusV
	arsR	arsR1	ArsR-family transcription regulator

### Genetic composition of *C. pseudotuberculosis* Pathogenicity Islands

The genetic composition of PAIs can shed light on the lifestyle of pathogenic bacteria, since they include virulence genes that mediate mechanisms of adhesion, invasion, colonization, proliferation into the host and evasion of the immune system [29], [30]. In addition, PAIs are characterized as being unstable regions that can be affected by insertions and deletions, influencing bacterial adaptability to new environments and hosts [31]. Here follows descriptions of the most relevant genetic elements found in the *C. pseudotuberculosis* pathogenicity islands. For more information, see the list of these orthologous genes in other *Corynebacterium* species in the Table S1 (online supporting information).

#### PiCp 1

*C. pseudotuberculosis* PiCp 1 harbors key genes involved in virulence and pathogenicity; these include PLD, the major virulence factor of this organism, which plays a role in spreading through the host; the *fag* operon, responsible for extracellular iron acquisition and, consequently, for survival in hostile environments; and a transposase gene, probably responsible for insertion of the island into the *C. pseudotuberculosis* genome. The finding that *C. ulcerans* can produce phospholipase D protein [32] indicates acquisition of PiCp1 by both *C. pseudotuberculosis* and *C. ulcerans*.

#### PiCp 2

Gene *mgtE* of island 2 has Mg^2+^ influx activity [33]. In prokaryotes, Mg^2+^ has been identified as an important regulatory signal that is essential for virulence, since it is involved in thermal adaptation, protecting bacteria from heat shock caused by fever in warm-blooded mammals [34]. Translation of the *mgtE* gene is regulated by changes in cytosolic Mg*^2+^* concentration; loss of MgtE reduces biofilm formation and motility in the pathogenic bacteria *Aeromonas hydrophila* [33].

The protein MalL (*malL*), a maltose-inducible α-glucosidase, hydrolyzes various disaccharides, such as maltose and isomaltose, which can serve as carbon and energy sources [35], [36].

The *tetA* gene codes for a tetracycline-efflux transporter protein that extrudes antibiotics from the cell and confers resistance to biofilm cells. The *tetA* gene is often carried by transmissible elements, such as plasmids, transposons, and integrons [37], thus explaining its presence in a PAI.

The *sigK* gene is an extracytoplasmic function sigma factor (sigma ECF) regulated by cskE, an anti-sigma factor. Another sigma ECF, *sigK*, mediates targeted alterations in bacterial transcription via transduction of extracellular signals. In *M. tuberculosis*, *sigK* regulates several genes (*Rv2871*, *mpt83*, dipZ, *mpt70*, *Rv2876*, and *mpt53*). Also, *sigK* mutations produce reduced quantities of the antigens MPT70 and MPT83 in vitro, and only induce strong expression during infection of macrophages [38]–[40].

PiCp2 also harbors a *dipZ* gene, which is regulated by *sigK* and seems to play a role in macrophage infection by *M. tuberculosis*, although its function is not clearly elucidated. DipZ is found as two separate proteins in most bacteria: CcdA and TlpA-like. Also, a full-length *dipZ* gene, found in the phylum *Actinobacteria*, is present exclusively in pathogenic bacteria (*C. diphtheriae*, *C. jeikeium*, *M. avium*, *M. kansasii*, *M. marinum*, *M. ulcerans* and *M. tuberculosis*) [40].

#### PiCp 3

*potG* gene, of the *pot*FGHI operon, is a membrane-associated/ATP-binding protein that provides energy for putrescine (polyamine) uptake from the periplasmic space [41]. Although the *potFGHI* operon is a putrescine-specific transport system, *potG* is downregulated by another polyamine (spermine), which is produced only by eukaryotes. Carlson et al. (2009) demonstrated that transcription of the *potG* gene in *Francisella tularensis* decreases with high levels of spermine, while transcription of IS elements ISFtu1 and ISFtu2 increases in response to high levels of spermine in macrophages responding to bacterial infection. Also, many of the upregulated genes of *F. tularensis* (pseudogenes and transposase genes) are located near the IS elements in the chromosome [42].

The gene *glpT* belongs to the organophosphate:phosphate antiporter family of the major facilitator superfamily (MFS); it mediates transport of glycerol 3-phosphate (G3P) across the membrane in bacteria [43].

The PhoPR system regulates expression of various genes involved in metabolic, virulence and resistance processes in several intracellular bacterial pathogens [44]. Based on the information obtained from the complete genome sequence of *C. pseudotuberculosis*, we found that the PhoPR system is constituted of the *phoP* (714 bp) and *phoR* (1506 bp) genes, separated by a small 39-bp sequence, suggesting that these two genes are transcribed by a bicistronic operon. The size and organization of this system in *C. pseudotuberculosis* is similar to those of other Gram-positive bacteria [45]. Live bacteria attenuated via *phoP* inactivation are also promising vaccine candidates against tuberculosis. Several studies have reported the efficacy of attenuated mutant strains of *M. tuberculosis* as vaccines [46], [47]. Phylogenetic relationships within the class *Actinobacteria* strongly suggest correlation of the *C. pseudotuberculosis* PhoPR system with virulence mechanisms. The *phoP* gene is an important subject for regulation studies; and is also a probable vaccine candidate against CL.

#### PiCp4

The operon *ciuABCDE* (*corynebacterium* iron uptake) was described in *C. diphtheriae* as an iron transport and siderophore biosynthesis system. Proteins involved in iron acquisition are recognized as virulence factors, since they help pathogens to obtain iron from a host by using siderophores to strip iron from carrier proteins, such as transferrin, lactoferrin, and hemoglobin-haptoglobin [48,48].

#### PiCp5

Island 5 harbors a gene (*pfoS*) related to the *pfoR* superfamily. The *pfo*R gene was previously characterized as responsible for positive regulation of production of perfringolysin A (*pfoA*) and other toxins in *Clostridium perfringens* [50]. The virulence factors regulated by *pfoR* have not been totally elucidated. However, it is well known that deactivation of this gene inhibits hemolysis through negative regulation of several *C. perfringens* toxins. *Clostridium perfringens* harbors a phospholipase C gene (*plc*) that serves a function similar to that of phospholipase D [51]. Additionally, PiCp 5 contains a putative sigma 70 factor that is responsible for transporting the transcription machinery to specific promoters. Interestingly, the putative sigma 70 factor presents a nonsense mutation in *C. pseudotuberculosis* strain C231, which could be responsible for differential gene expression.

#### PiCp6

The *pipA1* gene, which codes for a proline iminopeptidase, may have a role in pathogenesis, since it catalyses the removal of N-terminal proline residues from peptides; it also has a role in energy production [52]. In addition, a PIP-type protein is required for virulence of *Xanthomonas campestris pv. campes*tris [53].

#### PiCp7

Island 7 harbors a urease operon that is also present in *C. glutamicum*; it is flanked, on both sides, by regions that are absent in the non-pathogenic *C. glutamicum*. This mosaicism is a common feature of pathogenicity islands [54]. The *ure* operon presents a codon usage deviation in *C. glutamicum*, as in *C. pseudotuberculosis*, indicating that this region is a putative genomic island in *C. glutamicum*.

The *ure* operon is responsible for nitrogen acquisition through hydrolysis of urea to carbamate and ammonia. Production of ammonia by uropathogenic and enteropathogenic bacteria causes cellular damage and compromises the action of the host's immune system [55]. Considering this fact, due to the intramacrophagic location of *C. pseudotuberculosis* and the finding of this operon in a non-pathogenic bacterial species, additional studies will be needed to elucidate how *C. pseudotuberculosis* obtains urea from the host and how this operon affects pathogenicity.

PiCp 7 also harbors a lysyl-tRNA synthetase (*lysS*), responsible for lysine incorporation into its respective transfer tRNA. The importance of *lysS* would normally make its location on a PAI inviable, since it is essential for cell metabolism. However, it is the only tRNA synthetase gene that is duplicated in the genome.

### Protein classification of *C. pseudotuberculosis* in the biological process

Using the controlled vocabulary of functional terms proposed by the Gene Ontology (GO) Consortium for gene products classification [56], the predicted proteomes of the two genomes were analyzed according to the three organizing principles of gene ontology: cellular component, biological process and molecular function. The most abundantly represented categories are linked to metabolic processes in the two strains (cellular metabolic, biosynthetic, primary and macromolecule processes).

The gene products composition characterized using GO terminology suggests that *C. pseudotuberculosis* is a facultative intracellular pathogen. It is commonly found that pathogens specialized for an intracellular lifestyle have a high proportion of proteins linked to the above-mentioned processes. Moreover, the low proportion of proteins linked to the metabolism of secondary metabolites is an indication that *C. pseudotuberculosis* does not possess the metabolic machinery to deal with secondary metabolites, because they are supplied by the host.

### Sub-cellular localization of *C. pseudotuberculosis* proteins

Prediction of the sub-cellular localization of *C. pseudotuberculosis* proteins was made by *in silico* analysis, using the SurfG+ tool [57]. Surfg+ is a pipeline for protein sub-cellular prediction, incorporating commonly used software for motif searches, including SignalP, LipoP and TMHMM, along with novel HMMSEARCH profiles to predict protein retention signals. Surfg+ starts by searching for retention signals, lipoproteins, SEC pathway export motifs and transmembrane motifs, roughly in this order. If none of these motifs are found in a protein sequence, then it is characterized as being cytoplasmic. A novel possibility introduced by Surfg+ is the ability to distinguish between integral membrane proteins versus PSE (potentially surface-exposed proteins). This is done by a parameter that determines the expected cell wall thickness, expressed in amino acids. Using published information or electron microscopy, it is possible to estimate cell wall thickness value for procaryotic organisms. *C. pseudotuberculosis* proteins were classified into four different sub-cellular locations: cytoplasmic, membrane, PSE (potentially surface exposed), or secreted. The *C. pseudotuberculosis* genomes were compared to those of other species of the genus, including C. *diphtheriae, C. efficiens, C. glutamicum*, C. *jeikeium* and C. *urealyticum*, also predicted by Surfg+, based on published cell wall thicknesses. Table 3 shows the number of predicted proteins in each sub-cellular location.

**Table 3:** Subcellular prediction of the protein locations derived from complete genomes of *Corynebacterium* species.

Category/Species	Ce	CgB	CgK	CgR	Cj	Cd	Cu	Cp1002	CpC231	Total
Cytoplasm	2,158	2,11	2,082	2,158	1,49	1,594	1,432	1,399	1,389	15,812
Cytoplasm	504	557	541	561	333	375	332	364	356	3,923
PSE	230	254	249	252	197	204	179	201	201	1,967
Secreted	102	136	121	109	100	99	79	95	107	948
**Total**	2,994	3,057	2,993	3,08	2,12	2,272	2,02	2,059	2,053	22,648

Ce: *C. efficiens*; CgB: *C. glutamicum B*; CgK: *C. glutamicum K*; CgR: *C. glutamicum R*; Cj: *C. jeikeium;* Cd: *C. diphtheriae;* Cu: *C. urealyticum;* Cp1002: *C. pseudotuberculosis* 1002; CpC231: *C. pseudotuberculosis* C231. PSE: potential surface exposure.

Comparison of the frequencies of subcellular occurrence of the *C. pseudotuberculosis* proteins and other *Corynebacterium* proteomes was made with Chi-square tests. The ratio between the four groups (cytoplasmic, membrane anchored, potentially exposed and secreted proteins) was found to be nearly constant among the *Corynebacterium* species. The proportions of the four protein categories cited above were similar to published data [58], [59]. Song and colleagues (2009) showed that approximately 30% of proteins secreted in gram-positive bacteria are exported through the Sec pathway. Few proteins (n = 27) were predicted to be secreted by the Tat pathway in Cp1002. About 2% of the proteins predicted to be secreted presented tertiary structures. In terms of proportions of secreted proteins, Cp1002 and CpC231 are at the higher end of the spectrum. They present 4.61 and 5.21%, respectively, predicted secreted proteins (Table 3).

### Differences in metabolic pathways in the two strains of *C. pseudotuberculosis*

**Figure 2:**
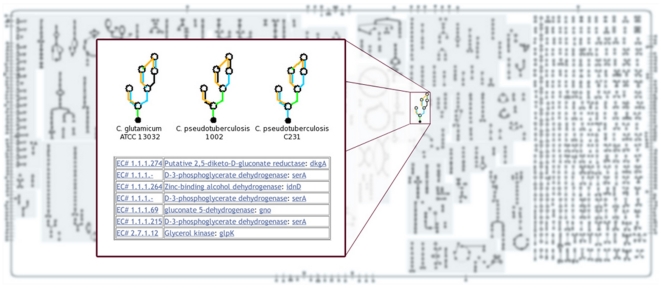
***Corynebacterium glutamicum*** metabolic pathways overview. *C. glutamicum* reactions are presented in blue and the reactions shared with *C. pseudotuberculosis* C231 and 1002 in red and green, respectively. By clicking on any compound or reaction, a window pops up showing details of each pathway. The fatty acid biosynthesis initiation pathway is the chosen example since computational evidence indicates it is not present only in strain C231.

Automated reconstruction of the *C. pseudotuberculosis* Cp1002 metabolic pathways identified 156 pathways and 744 enzymatic reactions. As expected, quite similar results were encountered for strain CpC231: 154 pathways and 754 reactions (Table 4). Proteins of predicted functions that did not map to pathways, such as transport reactions, enzymes, transporters, and compounds, were also identified. The metabolic pathway database can be accessed online at http://corynecyc.cebio.org. This database enabled us to visualize and compare the metabolism of these two *C. pseudotuberculosis* strains (Figure 2).

**Table 4:** Comparative summary of the *Corynebacterium pseudotuberculosis* strain gene data types.

Data Type	Cp1002	CpC231
Gene products	2,059	2,053
Pathways	156	154
Enzymatic Reactions	744	754
Transport Reactions	8	4
**Polypeptides**	2,065	2,059
Enzymes	516	506
Transporters	10	10
Compounds	639	651

We made a comparative analysis of transport reactions, pathways, compounds and proteins for *C. pseudotuberculosis* strains Cp1002 and CpC231 (Table 5). Despite the high similarity of the metabolic pathways, some differences were observed.

**Table 5:** Comparative summary of the number of pathways of *Corynebacterium pseudotuberculosis* strains Cp1002 and CpC231.

Pathway Class	Cp1002	CpC231
- Pathway subclass		
**Biosynthesis**	105	104
- Amine and Polyamine Biosynthesis	5	3
- Amino acid Biosynthesis	25	26
- Aminoacyl-tRNA Charging	1	1
- Aromatic Compound Biosynthesis	1	1
- Carbohydrate Biosynthesis	10	7
- Cell structure Biosynthesis	4	4
- Cofactor, Prosthetic Group, Electron Carrier Biosynthesis	27	29
- Fatty Acid and Lipid Biosynthesis	8	7
- Metabolic Regulator Biosynthesis	1	2
- Nucleoside and Nucleotide Biosynthesis	12	12
- Other Biosynthesis	1	1
- Secondary Metabolites Biosynthesis	1	2
**Degradation/Utilization/Assimilation**	53	54
- Alcohol Degradation	2	1
- Aldehyde Degradation	1	1
- Amine and Polyamine Degradation	5	4
- Amino Acid Degradation	11	12
- C1 Compound Utilization and Assimilation	4	4
- Carbohydrate Degradation	7	7
- Carboxylate Degradation	5	4
- Degradation/Utilization/Assimilation - Other	5	5
- Fatty Acid and Lipid Degradation	3	2
- Inorganic Nutrient Metabolism	4	6
- Nucleoside and Nucleotide Degradation and Recycling	2	3
- Secondary Metabolite Degradation	5	5
**Generation of precursor metabolites and energy**	16	19
**Total**	163	164

The metabolic pathways in each of the two bacterial strains (Cp1002 and CpC231) were classified into several pathway classes; each pathway class was further broken down to show the distribution of pathways among the next-level subclasses. Analysis of the metabolism database of *C. pseudotuberculosis* strains Cp1002 and CpC231 revealed specific pathway differences between the two strains. Overall, CpC231 had 13 specific metabolic pathways not found in strain Cp1002, and the latter had 11 metabolic pathways not found in strain CpC231 (Table 6).

**Table 6:** Table listing the *Corynebacterium pseudotuberculosis* strain-specific pathways.

Pathway Class	Cp1002	CpC231
Pathway Name		
**Biosynthesis - Amines and Polyamines Biosynthesis**		
choline degradation I	present	absent
glycine betaine biosynthesis I (Gram-negative bacteria)	present	absent
**Biosynthesis - Amino acid Biosynthesis**		
citrulline-nitric oxide cycle	absent	present
**Carbohydrates Biosynthesis**		
gluconeogenesis	present	absent
trehalose biosynthesis II	present	absent
trehalose biosynthesis III	present	absent
**Biosynthesis - Cofactor, Prosthetic Group, and Electron Carrier Biosynthesis**		
adenosylcobalamin biosynthesis from cobyrinate a,c-diamide I	absent	present
heme biosynthesis from uroporphyrinogen I	present	absent
heme biosynthesis from uroporphyrinogen II	absent	present
siroheme biosynthesis	absent	present
**Biosynthesis - Fatty Acid and Lipid Biosynthesis**		
biotin-carboxyl carrier protein	absent	present
cardiolipin biosynthesis I	present	absent
fatty acid biosynthesis initiation I	present	absent
**Secondary Metabolite Biosynthesis**		
canavanine biosynthesis	absent	present
**Biosynthesis - Metabolic Regulators Biosynthesis**		
citrulline-nitric oxide cycle	absent	present
**Degradation - Alcohols Degradation**		
glycerol degradation II	present	absent
**Degradation - Aldehyde Degradation**		
methylglyoxal degradation I	absent	present
methylglyoxal degradation III	present	absent
Degradation - Amine and Polyamine Degradation		
choline degradation I	present	absent
**Degradation - Amino Acid Degradation**		
2-ketoglutarate dehydrogenase complex	absent	present
citrulline-nitric oxide cycle	absent	present
valine degradation I	present	absent
**Degradation - Carboxylate Degradation**		
acetate formation from acetyl-CoA I	present	absent
**Degradation - Fatty Acid and Lipids Degradation**		
triacylglycerol degradation	present	absent
**Inorganic Nutrients Metabolism**		
nitrate reduction III (dissimilatory)	absent	present
nitrate reduction IV (dissimilatory)	absent	present
**Degradation - Nucleoside and Nucleotide Degradation and Recycling**		
purine deoxyribonucleoside degradation	absent	present
**Generation of precursor metabolites and energy**		
2-ketoglutarate dehydrogenase complex	absent	present
nitrate reduction III (dissimilatory)	absent	present
nitrate reduction IV (dissimilatory)	absent	present

Two amine and polyamine biosynthesis pathways, choline degradation I and glycine betaine biosynthesis I (Gram-negative bacteria), were found in strain Cp1002 but not in strain CpC231. Strain CpC231 was found to have an extra amino acid biosynthesis pathway, the citrulline-nitric oxide cycle. Strain Cp1002 was found to have three additional carbohydrate biosynthesis pathways: gluconeogenesis, trehalose biosynthesis II and trehalose biosynthesis III. Strain CpC231 showed three cofactor biosynthesis, prosthetic group and electron carrier pathways, corresponding to adenosylcobalamin biosynthesis from cobyrinate a,c-diamide I, heme biosynthesis from uroporphyrinogen II and siroheme biosynthesis. Strain Cp1002 showed only one unique cofactor biosynthesis pathway, heme biosynthesis from uroporphyrinogen I. Two extra pathways of fatty acid and lipid biosynthesis were found in strain Cp1002, cardiolipin biosynthesis I and fatty acid biosynthesis initiation I. Strain CpC231 showed only the biotin-carboxyl carrier protein. Among metabolic regulator biosynthesis genes, strain CpC231 showed the citrulline-nitric oxide cycle. Strain CpC231 also showed an extra pathway, the canavanine biosynthesis pathway, part of secondary metabolite biosynthesis.

Among degradation/utilization/assimilation pathways, strain Cp1002 showed an extra pathway: glycerol degradation II, for alcohol degradation, as well as choline degradation I for amine and polyamine degradation. Strain CpC231 was found to have two additional pathways, 2-ketoglutarate dehydrogenase complex and citrulline-nitric oxide cycle, for amino acid pathways; strain Cp1002 showed only one extra pathway, valine degradation I. Among carboxylate degradation pathways, involving fatty acid and lipid degradation, strain Cp1002 showed two extra pathways: one corresponding to acetate formation from acetyl-CoA I, and the second linked to triacylglycerol degradation. Two inorganic nutrient metabolism pathways were found in strain CpC231 but not in strain Cp1002: nitrate reduction III (dissimilatory) and nitrate reduction IV (dissimilatory), and a nucleoside and nucleotide degradation and purine deoxyribonucleoside recycling degradation pathway.

Finally, when we analyzed the generation of precursor metabolites and energy, strain CpC231 showed three extra pathways: 2-ketoglutarate dehydrogenase complex, nitrate reduction III (dissimilatory) and nitrate reduction IV (dissimilatory). The differences are presented in Table 6.

### Metabolic pathways in *C. pseudotuberculosis* compared to other *Corynebacterium* species

The web interface enabled us to visually compare the metabolic pathways of strains Cp1002 and CpC231 reactions (Figure 2) with those of four other bacteria of the genus *Corynebacterium*: *C. diphtheriae, C. efficiens, C. glutamicum, and C. jeikeium*. Using these diagrams we were able to easily spot reactions present in *C. pseudotuberculosis* and absent in other *Corynebacterium* species.

A comparative analysis of reactions, pathways, compounds and proteins was also done for *C. pseudotuberculosis* and other closely-related bacteria in the same genus. The list of *C. pseudotuberculosis* specific pathways is shown in Table 7.

**Table 7:** List of *Corynebacterium pseudotuberculosis* specific metabolic pathways that were compared to those of closely-related bacteria, including *C. diphtheriae*, *C. glutamicum*, *C. efficiens*, and *C. jeikeium*.

Pathway Class
Pathway Name
**Biosynthesis - Amino acid Biosynthesis**
Asparagine biosynthesis II
Lysine biosynthesis V
**Biosynthesis - Metabolic Regulators Biosynthesis**
Citrulline-nitric oxide cycle
**Biosynthesis - Nucleoside and Nucleotide Biosynthesis**
Salvage pathways of pyrimidine deoxyribonucleotides
**Degradation - Alcohol Degradation**
Glycerol degradation II
**Degradation - Aldehyde Degradation**
Methylglyoxal degradation III
**Degradation - Amino Acid Degradation**
Alanine degradation IV
Citrulline-nitric oxide cycle
Lysine degradation I
**Degradation - C1 Compound Utilization and Assimilation**
Reductive monocarboxylic acid cycle
Degradation - Carbohydrate Degradation
Chitobiose degradation
**Degradation - Carboxylate Degradation**
Conversion of succinate to propionate
**Degradation - Fatty Acid and Lipid Degradation**
Phospholipases
**Inorganic Nutrients Metabolism**
Ammonia oxidation I (aerobic)
Nitrate reduction IV (dissimilatory)
**Degradation - Secondary Metabolite Degradation**
D-glucarate degradation
Betanidin degradation
D-galactarate degradation
**Generation of precursor metabolites and energy**
Ammonia oxidation I (aerobic)

We found that *C. pseudotuberculosis* has several pathways that are not found in other species of the genus *Corynebacterium*. However, little information is available about these pathways in *Corynebacterium* spp. We found no published information concerning the following pathways: asparagine biosynthesis II, citrulline-nitric oxide cycle (amino acid biosynthesis and degradation), pyrimidine deoxyribonucleotide salvage pathways, methylglyoxal degradation III, reductive monocarboxylic acid cycle, chitobiose degradation, conversion of succinate to propionate, ammonia oxidation I (aerobic), nitrate reduction IV (dissimilatory), D-glucarate degradation, betanidin degradation, D-galactarate degradation, and ammonia oxidation I (aerobic).

Some studies reported five pathways: lysine biosynthesis V, glycerol degradation II, alanine degradation IV, lysine degradation I and phospholipases. However, none of the studies, except for those concerning lysine degradation I and phospholipase pathways, involved *C. pseudotuberculosis*. Most of these studies were carried out with *C. glutamicum*.

Four papers concerning *C. glutamicum* were found for the lysine degradation I pathway [60]–[63]. Studies have focused on: acetohydroxyacid synthase, a novel target for improvement of L-lysine production [62], improvement of L-lysine formation by expression of the *Escherichia coli* pntAB genes [61], genetic and functional analysis of soluble oxaloacetate decarboxylase [63], and modeling and experimental design for metabolic flux analysis of lysine-producing *Corynebacteria* by mass spectrometry [64].

Six studies were found concerning the glycerol degradation II pathway, one performed with C. *diphtheria* [65] and four with C. *glutamicum* [66]–[69]. In the sixth study, made with *C. glutamicum*, we found information on the alanine degradation IV pathway [64].

Approximately 140 studies, of which 107 were made with *C. glutamicum* alone, dealt with the lysine degradation I pathway, in which cadaverine is biosynthesized from L-lysine. Cadaverine is reported to be essential for the integrity of the cell envelope and for normal growth of the organism, as well as for inhibiting porin-mediated outer membrane permeability, thereby protecting cells from acid stress [70], [71].

All studies of specific phospholipase pathways were carried out with *C. pseudotuberculosis*. Phospholipases hydrolyze phospholipids and are ubiquitous in all organisms. Several types of phospholipases were reported; phospholipase D is the best studied and has been considered a major virulence factor for *C. pseudotuberculosis* [72], [73]. In our analyses, none of the five bacteria of the genus *Corynebacterium* were found to have pathways belonging to the following subclasses: siderophore biosynthesis; chlorinated compound degradation; cofactor, prosthetic group, electron carrier, and hormone degradation. Clearly more biochemical studies are needed. Our current study brings new insight to relevant biochemical pathways that can be further explored experimentally.

We made a comparative summary of the metabolic pathways of *C. pseudotuberculosis* strains Cp1002 and CpC231 and *C. glutamicum* (Table 8). *C. glutamicum* has several metabolic pathways not found in *C. pseudotuberculosis* Cp1002 and/or in *C. pseudotuberculosis* CpC231. Overall, *C. glutamicum* has approximately 40 additional metabolic pathways.

**Table 8:** Comparative summary of *Corynebacterium pseudotuberculosis* strains Cp1002 and CpC231 and *C. glutamicum* pathways.

Pathway Class	Cp1002	CpC231	*C. glutamicum*
- Pathway subclass			
**Biosynthesis**	105	104	131
- Amine and Polyamine Biosynthesis	5	3	3
- Amino acid Biosynthesis	25	26	29
- Aminoacyl-tRNA Charging	1	1	3
- Aromatic Compound Biosynthesis	1	1	1
- Carbohydrate Biosynthesis	10	7	9
- Cell structure Biosynthesis	4	4	4
- Cofactor, Prosthetic Group, Electron Carrier Biosynthesis	27	29	38
- Fatty Acid and Lipids Biosynthesis	8	7	14
- Metabolic Regulator Biosynthesis	1	2	1
- Nucleoside and Nucleotide Biosynthesis	12	12	10
- Other Biosynthesis	1	1	1
- Secondary Metabolite Biosynthesis	1	2	6
**Degradation/Utilization/Assimilation**	53	54	72
- Alcohols Degradation	2	1	2
- Aldehyde Degradation	1	1	1
- Amine and Polyamine Degradation	5	4	6
- Amino Acid Degradation	11	12	15
- Aromatic Compound Degradation	0	0	9
- C1 Compound Utilization and Assimilation	4	4	2
- Carbohydrate Degradation	7	7	10
- Carboxylate Degradation	5	4	6
- Chlorinated Compound Degradation	0	0	4
- Degradation/Utilization/Assimilation - Other	5	5	2
- Fatty Acid and Lipid Degradation	3	2	2
- Inorganic Nutrient Metabolism	4	6	9
- Nucleoside and Nucleotide Degradation and Recycling	2	3	1
- Secondary Metabolite Degradation	5	5	4
**Generation of precursor metabolites and energy**	16	19	25
**Total**	163	164	206

Among biosynthesis pathways, *C. glutamicum* showed around 30 extra pathways when compared to the two strains of *C. pseudotuberculosis*. These involve pathways of amino acid biosynthesis, aminoacyl-tRNA charging, cofactors, prosthetic groups, electron carrier biosynthesis, fatty acid and lipid biosynthesis and secondary metabolite biosynthesis. However, the two strains of *C. pseudotuberculosis* also have specific pathways that were not found in *C. glutamicum*, these being the pathways of amine and polyamine biosynthesis, carbohydrate biosynthesis and nucleoside and nucleotide biosynthesis.

Among the degradation/utilization/assimilation pathways, *C. glutamicum* presented around 20 extra pathways, when compared to *C. pseudotuberculosis* Cp 1002 and *C. pseudotuberculosis* CpC231. These specific pathways of *C. glutamicum* correspond to pathways of amine and polyamine degradation, amino acid degradation, aromatic compound degradation, carbohydrate degradation, carboxylate degradation, chlorinated compound degradation and the metabolism of inorganic nutrients. Again, the two strains of *C. pseudotuberculosis* also had specific pathways involving degradation/utilization/assimilation, fatty acid and lipid degradation and secondary metabolite degradation that were not found in *C. glutamicum*.

We found 25 pathways involving generation of precursor metabolites and energy in *C. glutamicum*, while *C. pseudotuberculosis* Cp1002 had only 16 and *C. pseudotuberculosis* CpC231 had 19.

## Discussion

### General aspects of the *C. pseudotuberculosis* genome

The *C. pseudotuberculosis* genome has proven to be one of the smallest genomes of the *Corynebacterium* genus sequenced so far, with Cp1002 being the smallest and Cp231 the fourth smallest, larger only than Cp1002, *C. lipophiloflavum* DSM 44291 (2,293,743 bp) and *C. genitalium* ATCC 33030 (2,319,774 bp); the latter two are both human pathogens. *Corynebacterium pseudotuberculosis* has a very small genetic repertoire, with considerable gene loss when compared to non-pathogenic species such as *C. glutamicum* and *C. efficiens*. When predicted proteomes were compared, *C. pseudotuberculosis* showed a loss of approximately 1,220 genes, in comparison with *C. glutamicum*. Classification of these proteins using GO terminology showed that the majority are linked to metabolic processes, such as cellular, primary, biosynthetic, macromolecule, nitrogen compound and oxidation reduction processes.

Other characteristics of the *C. pseudotuberculosis* genome include the lowest GC content in the *Corynebacterium* genus, this being 52% in both the goat and sheep strains, followed by *C. diphtheriae* with a GC content of 53%. This contrasts with *C. urealyticum*, which has a GC content of 64%. Furthermore, *C. pseudotuberculosis* has a higher number of predicted pseudogenes and a lower number of tRNAs, when compared to other species of the *Corynebacterium* genus for which genome sequences are available.

Merjeh et al. (2009) made a comparative analysis of 317 genomes of bacteria with different lifestyles (free-living, facultative intracellular and obligate intracellular). They found evidence that peculiar characteristics in bacterial genomes can drive the organisms to certain lifestyles. All characteristics cited in their work were identified in the *C. pseudotuberculosis* genomes. Lower GC content generally can occur due to gene loss, which is a means to contract the genome in response to a specialized environment. Moreover, presence of a higher number of pseudogenes could be evidence of bacterial mechanisms to generate non-functional genes and subsequent gene loss [19]. In addition, the high proportion of proteins linked to primary metabolism, and the small proportion of proteins related to secondary metabolism, is usually seen in facultative intracellular organisms. Taking these aspects of the genomic architecture of *C. pseudotuberculosis* into account, it can be affirmed that *C. pseudotuberculosis* has a facultative intracellular lifestyle.

### High similarity in the genome architecture

Usually, pseudogenes are characterized as genes that have lost their function in the genome, due either to changes in the reading frame (frameshifts) or to a premature stop codon. Pseudogenes are common in prokaryotes; most have been linked to a sudden change in the environment of the pathogen, with simultaneous loss of metabolic and respiratory activities [74].

The high number of pseudogenes in these two strains of *C. pseudotuberculosis* (52 in Cp1002 and 50 pseudogenes in CpC231) suggest an evolutionary process involving a contracting genome in this species. An example of this is also seen in *Mycobacterium leprae*, which has a large number of pseudogenes (around 1,000). When we compare *M. leprae* to *M. tuberculosis*, the latter has both considerably fewer genes and a higher number of pseudogenes that can drive this gene loss.

### Virulence factors acquired

Identification of pathogenicity islands (PAIs) in pathogenic bacteria is highly relevant for understanding the reasons behind different responses to vaccines and the biological mechanisms leading to genome plasticity. The biovars *equi* and *ovis* of *C. pseudotuberculosis* cause distinct diseases in their hosts; assessment of virulence genes could help identify genes involved in these host-specific differences.

Virulence genes, which are central to distinguishing pathogenic from non-pathogenic species, are present in PAIs in large numbers. Additionally, the fact that PAIs are a consequence of horizontal transfer events indicates that the virulence factors they contain can help increase the adaptability of strains to different host environments. This increase in adaptability is demonstrated by the finding of genes with functions associated with uptake of iron (*fag* operon), carbon (*malL*) and Mg^2+^ from the host, since this uptake improves survival under stress conditions, such as iron depletion, starvation and heat shock. Furthermore, PAIs of *C. pseudotuberculosis* present genes that respond to a macrophagic environment (*potG*, *sigK* and *dipZ*), which sheds new light on the mechanisms responsible for the intramacrophagic lifestyle of this organism.

### Gene Sharing among *C. pseudotuberculosis* strains

**Figure 3:**
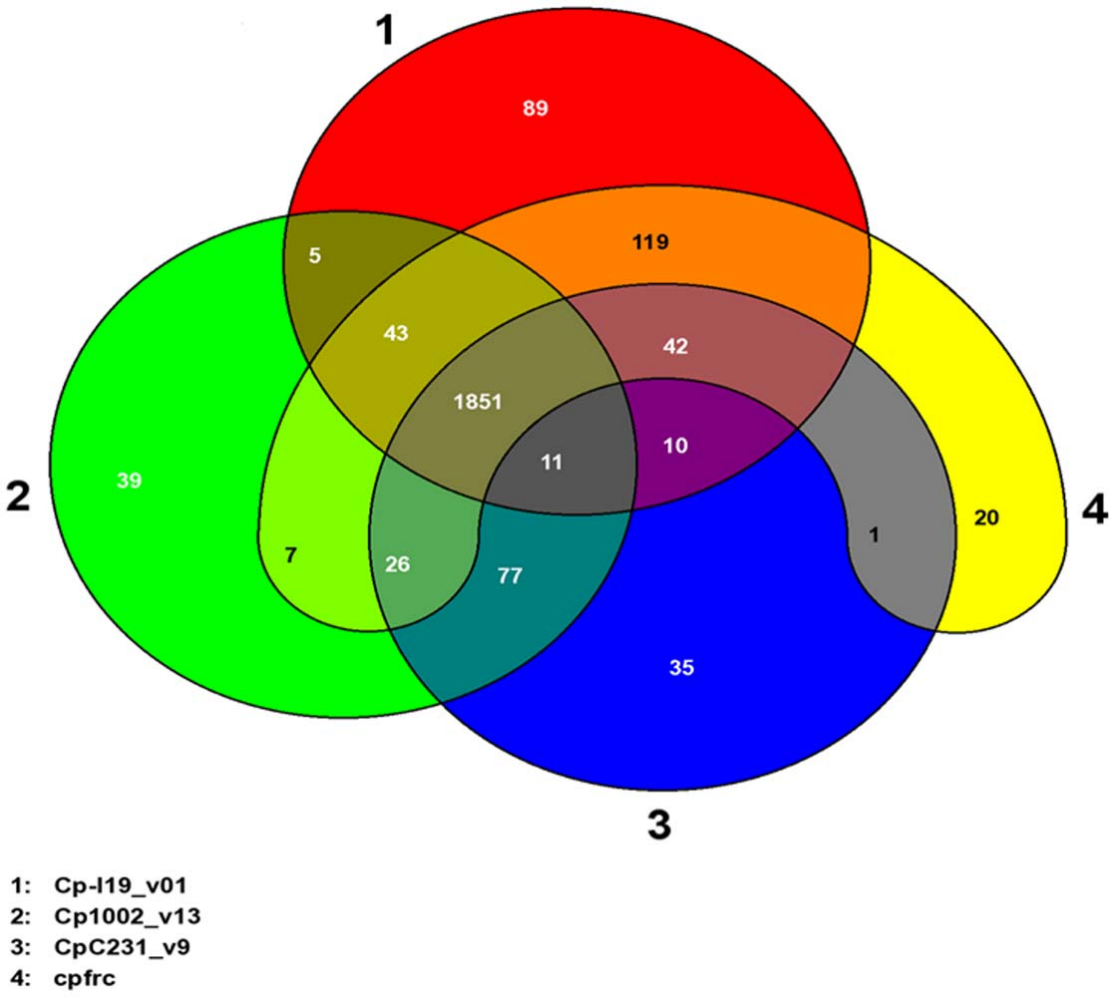
Venn diagram illustrating the three genomic categories of four *Corynebacterium pseudotuberculosis* strains: core, accessory and extended genome. Data obtained from the comparison of the predicted proteomes of four *C. pseudotuberculosis* speices in the EDGAR program (Blom et al., 2009). In red: Cp-I19; green: Cp1002; blue: CpC231 and yellow: CpFRC41. The remaining colors illustrate the shared genes among strains. The numbers within the forms indicate the number of shared genes.

Considering the four available genomes of *C. pseudotuberculosis* strains (Cp1002, CpC231, and CpI19 pFRC41), we identified 1,851 whole genes shared among them (Figure 3).

This repertoire of genes is vast for this specie, since, among the four isolates the maximum number of genes is 2,377 (called the pangenome of the species). When we compare the number of genes shared by these four *C. pseudotuberculosis* strains with a study of 17 strains of the bacterium *E. coli* [75], we conclude that *C. pseudotuberculosis* has a greater proportion of shared genes. In isolates of *E. coli*, 2,220 genes constituted the core genome, less than half of the genes in this species, with a mean of 5,000 genes in each genome [75]. Other significant information that emerges from this data is that the *C. pseudotuberculosis* genomes are extremely similar, since we found no significant change in the composition of the repertoire of genes for this species after adding the two new strains (Figure 3).

### Gene Sharing between *C. pseudotuberculosis* and other *Corynebacterium* species

Previous comparative studies of sequences of the rpoB gene of *C. pseudotuberculosis* and *C. diphtheriae* have suggested a close relationship between them [27], [76]. In our current study, we confirmed this close relationship with several types of evidence: i) a similar codon bias, ii) high similarity at the amino acid level and iii) conserved synteny. Synteny analysis of the genomes of the two *C. pseudotuberculosis* strains compared to *C. diphtheriae* indicates that these genomes are highly conserved; the gene position is conserved within the species. This observation reinforces the conclusions of previous research claiming conserved synteny in this genus, which indicated that few rearrangement events occurred during evolution [25].

*Corynebacterium pseudotuberculosis* shares more orthologous genes with *C. glutamicum* (1,345 genes), *C. efficiens* (1,330), *C. diphtheriae* (1,263 genes) and *C. auricumucosum* (1,273 genes); it shares only 1,030 genes with *C. jeikeium* and *C. kroppenstedtii*.

The larger number of genes shared between *C. pseudotuberculosis*, *C. glutamicum* and *C. efficiens* (72%), compared to other species (pathogenic species, 60%), may be a result not only of their close relationships, but also because a comparison is made among species with a larger gene repertoire, such as *C. glutamicum* and *C. efficiens*, which are non-pathogenic microorganisms, thus increasing the possibility of sharing genes.

### Lineage-specific genes in *C. pseudotuberculosis*

Most of the lineage-specific genes are involved in processes of virulence, pathogenicity, drug resistance and response to certain types of stress. These factors can increase the adaptability of microorganisms to the niches they inhabit, but they are not indispensable to the survival of pathogens. Moreover, some copies of these genes can be acquired by horizontal transfer. These genes are not ORFans; they already have been characterized in other species. The terminology ‘lineage-specific’ portrays only some genes found among the four strains in our study; the same genes may be found in other species.

We found 49 lineage-specific genes in CpC231 and 52 in Cp1002. For most of them, we did not have a descriptive characterization of their products, and they were classified as hypothetical proteins. In addition, many of these identified genes, in both strains, encode membrane and secreted proteins and pseudogenes. On the other hand, some well-characterized proteins were found in the genome. One example is found in CpC231, which has the gene called *pth*A; this gene encodes an effector system of type III secretion and is related to bacterial growth and host cell lesions, as found in *Xanthomonas campestris* [77]. This gene may be a good target for understanding the development of *C. pseudotuberculosis* CpC231 inside the host and the necrosis seen in CL abscesses, where it plays the same role in this pathogen.

In Cp1002, a very interesting gene was found, *tat*A, which encodes a membrane protein translocase, involved in the secretion of proteins in their final conformation, through the inner membrane to the extracellular environment. This gene is interesting because it is independent of the Sec secretion system and is a unique copy among the strains, suggesting that Cp1002 may have other routes for secretion. Regarding the large number of hypothetical proteins found in this strain, it may harbor genes that came from horizontal transfer, including some from phylogenetically-distant organisms, for which genomic molecular characterization has not been made.

Finally, lineage-specific genes may be good tools for understanding the host-pathogen interaction and may be good targets for the development of computational tools for differentiation between these strains, for molecular epidemiology.

### Biochemical properties of *C. pseudotuberculosis*

In the latest review of the biochemical properties of *C. pseudotuberculosis* [76], Dorella and colleagues gathered information concerning its metabolism, virulence and pathogenesis. They reported that the peptidoglycan in the cell wall is based on meso-DAP acid, and that arabinose and galactose are major cell-wall sugars. Our analyses predicted all of the reactions of the peptidoglycan biosynthesis II pathway; the meso-DAP acid compound was found as a product/substrate of the reaction catalyzed by UDP-N-acetylmuramyl tripeptide synthase (6.3.2.13). The complete pathway of UDP-galactose biosynthesis was also found; although there was no evidence of biosynthesis of arabinose, we detected a membrane transporter, known as arabinose efflux permease.

We also found short-chain mycolic acids; 10 variations of acids of this type were encountered, including 6-O-cis-keto-mycolyl-trehalose-6-phosphate, and 6-O-mycolyl-trehalose-6-phosphate. The two strains of *C. pseudotuberculosis* showed considerable fermentation ability, with several fermentation pathways, including glycolysis III, mixed acid fermentation and pyruvate fermentation to acetate IV, ethanol I and lactate.

Several sugar degradation pathways were also found in the two strains of *C. pseudotuberculosis*, including galactose, lactose, sucrose and L-and D-arabinose degradation. We confirmed that, as reported by Dorella et al. (2006), all these pathways produce acids and no gasses, generating large amounts of energy.

It was also previously reported that *C. pseudotuberculosis* is phospholipase D and catalase positive. Our analysis showed that both phospholipase D and catalase are involved in important processes. The main molecular functions of phospholipase D are phospholipase D activity, magnesium ion binding, NAPE-specific phospholipase D activity and sphingomyelin phosphodiesterase D activity. Catalase, which is produced by the *cat* gene, is involved in response to oxidative stress and oxidation reduction. Although two enzymes of the denitrification pathway (nitrate reduction I) were found, absence of the remaining enzymes is probably the determining factor for the inability of these strains to reduce nitrate to N^2^, as reported by Dorella et al. (2006).

We also detected iron acquisition genes (*fag*) A, B, C and D in both strains of *C. pseudotuberculosis* [78]. Genes *fagA* and *fagB* produce the integral membrane proteins FagA, an iron-enterobactin transporter, and FagBy; both have important roles, including ion, transmembrane, organic acid and protein transport. The ATP binding cytoplasmic membrane protein, FagC, produced by gene *fagC*, has two main molecular functions: ATP binding and ATPase activity. Finally, gene *fagD* produces the iron siderophore binding protein, FeAcquisition gene D, which has a role in iron ion transmembrane transport activity.

Computational reconstruction of the *C. pseudotuberculosis* pathways in our database not only allowed us to better visualize the metabolism of this bacterium, but also to compare it to closely related species. The main purpose of this analysis was to describe *C. pseudotuberculosis* metabolism by computational means, providing a predictive tool for “wet-lab” research.

## Methods

### Bacterial strains and growth conditions

*Corynebacterium pseudotuberculosis* 1002 biovar ovis (herein referred to as Cp1002) is a wild strain, isolated from a caprine host in Brazil. *Corynebacterium pseudotuberculosis* C231 biovar ovis (herein referred to as CpC231) is also a wild strain, isolated from an ovine host in Australia. Both strains were confirmed to be *C. pseudotuberculosis* by routine biochemical tests (API CORYNE, Biomerieux, Marcy l'Etoile, France). These strains were maintained in brain-heart-infusion broth (BHI – HiMedia Laboratories Pvt. Ltda, India) at 37°C, under rotation.

### Preparation of high molecular weight DNA

Chromosomal DNA extraction was performed as follows: 50 mL of 48–72 h cultures of the two strains were centrifuged at 4°C and 2000 x *g* for 20 min. Cell pellets were re-suspended in 1 mL Tris/EDTA/NaCl [10 mM Tris/HCl (pH 7.0), 10 mM EDTA (pH 8.0), and 300 mM NaCl] and centrifuged again under the same conditions. Supernatants were discarded, and the pellets were re-suspended in 1 mL TE/lysozyme [25 mM Tris/HCl (pH 8.0), 10 mM EDTA (pH 8.0), 10 mM NaCl, and 10 mg lysozyme/mL]. Samples were then incubated at 37°C for 30 min. Thirty milliliters of 30% (w/v) sodium N-lauroyl-sarcosine (Sarcosyl) were added to each sample and the mixtures were incubated for 20 min at 65°C, followed by incubation for 5 min at 4°C. DNA was purified using phenol/chloroform/isoamyl alcohol (25∶24∶1) and precipitated with ethanol. DNA concentrations were determined spectrophotometrically, and the DNA was visualized in ethidium bromide-stained 0.7% agarose gels.

### Construction of *Corynebacterium pseudotuberculosis* genomic libraries and Sanger sequencing

For the shotgun strategy used to sequence *C. pseudotuberculosis* 1002, four small fragment libraries were constructed using the TOPO Shotgun cloning kit and the pCR4 Blunt-TOPO vector (Invitrogen), according to the manufacturer's instructions. Sanger sequencing was carried out using the Minas Gerais Genome Network (http://rgmg.cpqrr.fiocruz.br). A total of 6,144 forward and reverse reads were produced using the DYEnamic Dye Terminator kit and run in a Megabace 1000 automated sequencer (GE Healthcare).

### Genome Sequencing

Cp1002 was sequenced using both Sanger and pyrosequencing technologies. Pyrosequencing was carried out using 454 Life Sciences (Branford, CT). A total of 397,147 high quality reads and 86,154,153 high quality bases were obtained, which translates into approximately 31-fold coverage. The average length of the sequences was 253 bases. The sequences were delivered after quality filtering and preassembly with the Newbler assembler (454 Life Sciences).

CpC231 was sequenced with a Roche-454 FLX sequencer at the Australian Animal Health Laboratory, Geelong, Australia. A total of 347,361 reads generated 80,336,550 bases, giving 34-fold coverage of the genome. *De novo* assembly of the filtered sequence data was carried out using the Newbler software. This assembly produced 10 large contigs in four scaffolds. The remaining gaps in the genomic sequence were closed by PCR walking and Sanger sequencing of the resulting fragments.

### Treatment and assembly data

The raw Sanger data obtained from sequencing were processed using the Phred-Phrap-Consed package [75]. Possible contaminants (plasmid DNA, sequences with similarity to vectors and other contaminants) were discarded using the Cross_match program (www.phrap.org). The quality value used in the base-calling program was Q = 40 (Probability of incorrect base call 1 in 10,000/base call accuracy 99.99%). An assembly using Phrap parameters (Force Level: 40 and Gap Length: 10,000) was carried out.

The 454 data were processed using the Newbler assembler (454 Life Sciences), and the final genomic consensus sequence was obtained using the Phrap algorithm.

### Genome annotation

The annotation procedures involved the use of several algorithms in a multi-step process. Structural annotation was performed using the following software: FgenesB: gene predictor (www.softberry.com); RNAmmer: rRNA predictor [79]; tRNA-scan-SE: tRNA predictor [80]; and Tandem Repeat Finder: repetitive DNA predictor (tandem.bu.edu/trf/trf.html). Functional annotation was performed by similarity analyses, using public databases and InterProScan analysis [81]. Manual annotation was performed using Artemis [82].

Identification and confirmation of putative pseudogenes in the genome was carried out using Consed. Manual analysis was performed based on the Phred quality of each base in the frameshift area. This analysis enabled the identification of erroneous insertions or deletions of bases in the genome information produced by the sequencing process, and it avoided identification of false-positive pseudogenes.

Predictions of the cellular locations of *Corynebacterium* proteins were made using the program SurfG Plus (version 1.0), with a minimum protein size of 73 amino acids. Classification of predicted proteins in functional categories was made using the BLAST2GO program (www.blast2go.org). The cutoff value used was 10−6 (http://www.blast2go.org/).

### *In silico* Identification of Pathogenicity Islands

In order to accurately identify and classify putative Pathogenicity Islands (PAIs) in the corynebacterial genomes, we developed a combined computational approach using several in-house scripts to integrate the prediction of diverse algorithms and databases, namely: Colombo-SIGIHMM [83], Artemis [82], tRNAscan-SE [80]; EMBOSS-geecee [84], ACT: the Artemis Comparison Tool [85], and mVIRdb [86].

### *In silico* metabolic pathway construction

The two main data sources used for reconstructing the *C. pseudotuberculosis* metabolic pathways were the genome sequence file in FASTA format and the genome annotation file in GBK format. Metabolic pathways databases for strains 1002 and C231 were created using the Pathway tools 13 software, developed by SRI International [87]. The Pathway tools software contains algorithms that predict metabolic pathways of an organism from its genome by comparison to a reference pathways database known as MetaCyc [88]. Construction of a metabolic pathways database was done using BioCyc [89], in order to compare the different bacteria, *C. diphtheriae* NCTC 13129, *C. efficiens* YS-314, *C. glutamicum* ATCC 13032, and *C. jeikeium* K411, to the deduced *C. pseudotuberculosis* pathways.

### Comparative analysis of *Corynebacterium pseudotuberculosis* strains

Comparative analyses were made for the two *C. pseudotuberculosis* strains. Similarity analyses of the two genomes were made using the BLAST - NCBI [90], [91] and InterProScan databases. The Mauve algorithm (gel.ahabs.wisc.edu/mauve) and the ACT tool were used to identify whether blocks had undergone gene rearrangements or remained preserved. The Plotter program of the MUMMer 3.22 package (mummer.sourceforge.net) was used for synteny analysis.

## Supporting Information

Table S1 Orthologous genes present inside PAIs regions of *C. pseudotuberculosis* and their counterparts in other Corynebacterium species.
(DOC)
