# Supplementary material for: Evidence for Reductive Genome Evolution and Lateral Acquisition of Virulence Functions in Two Corynebacterium pseudotuberculosis Strains
Source: PLoS One. 2011 Apr 18;6(4):e18551. doi: 10.1371/journal.pone.0018551 (PMC3078919; doi:10.1371/journal.pone.0018551)
Supplement: Table S1 — Orthologous genes present inside PAIs regions of C. pseudotuberculosis and their counterparts in other Corynebacterium species. (DOC) [file pone.0018551.s001.doc]

**Table S1:** Orthologous genes present inside PAIs regions of *C. pseudotuberculosis* and their counterparts in other *Corynebacterium* species.

| Genes | *C. pseudotuberculosis 1002^a^* | *C. pseudotuberculosis C231^b^* | *C. pseudotuberculosis FRC41^c^* | *C. pseudotuberculosis I19^d^* | *C. aurimucosum^e^* | *C. diphtheriae^f^* | *C. jeikeium^g^* | *C. kroppenstedtii^h^* | *C. urealyticum^i^* |
| --- | --- | --- | --- | --- | --- | --- | --- | --- | --- |
| **PAI1 genes** |  |  |  |  |  |  |  |  |  |
|  | Cp1002_0022 | CpC231_0020 | ----- | CpI19_0022 | ----- | ----- | ----- | ----- | ----- |
|  | Cp1002_0023 | CpC231_0021 | cpfrc_00022 | CpI19_0023 | ----- | ----- | ----- | ----- | ----- |
|  | Cp1002_0024 | CpC231_0022 | cpfrc_00026 | ----- | ----- | ----- | ----- | ----- | ----- |
|  | Cp1002_0025 | CpC231_0023 | cpfrc_00027 | CpI19_0025 | ----- | DIP2208 | ----- | ----- | ----- |
| **tnp7109-9** | Cp1002_0026 | CpC231_0024 | cpfrc_00028 | CpI19_0026 | cauri_0198 | ----- | jk2029 | ----- | cu0513 |
| **pld** | Cp1002_0027 | CpC231_0025 | cpfrc_00029 | CpI19_0027 | ----- | ----- | ----- | ----- | ----- |
| **fagC** | Cp1002_0028 | CpC231_0026 | cpfrc_00030 | CpI19_0028 | cauri_0721 | DIP1059 | jk1818 | ckrop_0466 | cu0335 |
| **fagB** | Cp1002_0029 | CpC231_0027 | cpfrc_00031 | CpI19_0029 | cauri_2323 | DIP1060 | jk1817 | ckrop_0467 | cu0336 |
| **fagA** | Cp1002_0030 | CpC231_0028 | cpfrc_00032 | CpI19_0030 | cauri_2324 | DIP1061 | jk1816 | ckrop_0468 | cu0337 |
| **fagD** | Cp1002_0031 | CpC231_0029 | cpfrc_00033 | CpI19_0031 | cauri_2325 | DIP1062 | jk1776 | ckrop_0465 | cu0332 |
| **PAI2 genes** |  |  |  |  |  |  |  |  |  |
| **mgtE** | Cp1002_0046 | CpC231_0044 | cpfrc_00048 | CpI19_0046 | cauri_2131 | ----- | ----- | ----- | cu1056 |
| **malL** | Cp1002_0047 | CpC231_0045 | cpfrc_00049 | CpI19_0047 | cauri_0565 | DIP0533 | jk0778 | ckrop_1713 | ----- |
|  | Cp1002_0048 | CpC231_0046 | cpfrc_00050 | CpI19_0048 | cauri_1152 | ----- | jk0049 | ----- | ----- |
| **tetA** | Cp1002_0049 | CpC231_0047 | cpfrc_00051 | CpI19_0049 | ----- | ----- | ----- | ----- | ----- |
|  | Cp1002_0050 | CpC231_0048 | ----- | CpI19_0050 | ----- | ----- | ----- | ----- | ----- |
| **cskE-sigK** | Cp1002_0051 | CpC231_0049 | cpfrc_00052 | CpI19_0533 | cauri_0517 | DIP0099 | jk0025 | ckrop_0643 | cu0493 |
|  | Cp1002_0052 | CpC231_0051 | cpfrc_00054 | CpI19_0052 | ----- | ----- | ----- | ----- | ----- |
| **dipZ** | Cp1002_0053 | CpC231_0052 | cpfrc_00055 | CpI19_0053 | cauri_0515 | DIP0101 | jk0023 | ----- | ----- |
|  | Cp1002_0054 | CpC231_0053 | cpfrc_00056 | CpI19_0054 | ----- | ----- | ----- | ----- | ----- |
| **PAI3 genes** |  |  |  |  |  |  |  |  |  |
|  | Cp1002_0173 | CpC231_0176 | cpfrc_00175 | CpI19_0175 | cauri_0194 | DIP0281 | jk1994 | ckrop_0185 | cu1884 |
|  | Cp1002_0174 | CpC231_0177 | ----- | CpI19_0176 | ----- | ----- | ----- | ----- | ----- |
|  | Cp1002_0175 | CpC231_0178 | cpfrc_00176 | CpI19_0177 | ----- | ----- | ----- | ----- | ----- |
|  | Cp1002_0176 | CpC231_0179 | ----- | CpI19_0178 | ----- | ----- | ----- | ----- | ----- |
|  | Cp1002_0177 | CpC231_0180 | ----- | CpI19_0179 | ----- | ----- | ----- | ----- | ----- |
|  | Cp1002_0178 | CpC231_0181 | cpfrc_00177 | CpI19_0180 | ----- | ----- | ----- | ----- | ----- |
|  | Cp1002_0179 | CpC231_0182 | cpfrc_00178 | CpI19_0181 | ----- | ----- | ----- | ----- | ----- |
| **potG** | Cp1002_0180 | CpC231_0183 | cpfrc_00179 | CpI19_0182 | cauri_0570 | DIP0282 | jk0654 | ckrop_1658 | cu0462 |
| **afuB** | Cp1002_0181 | CpC231_0184 | cpfrc_00180 | CpI19_0183 | ----- | DIP0283 | ----- | ----- | ----- |
| **afuA** | Cp1002_0182 | CpC231_0185 | cpfrc_00181 | ----- | ----- | DIP0284 | ----- | ----- | ----- |
| **glpT** | Cp1002_0183 | CpC231_0186 | cpfrc_00182 | CpI19_0185 | ----- | DIP0285 | ----- | ckrop_0413 | ----- |
| **phoB** | Cp1002_0184 | CpC231_0187 | cpfrc_00183 | CpI19_0186 | cauri_2191 | DIP0286 | jk0341 | ckrop_0362 | cu0480 |
| **lcoS** | Cp1002_0185 | CpC231_0188 | cpfrc_00184 | CpI19_0187 | cauri_2190 | DIP0287 | jk0342 | ckrop_0363 | cu1441 |
|  | Cp1002_0186 | CpC231_0189 | cpfrc_00185 | CpI19_0188 | ----- | DIP0288 | jk1991 | ckrop_2029 | ----- |
| **PAI4 genes** |  |  |  |  |  |  |  |  |  |
|  | Cp1002_0980 | CpC231_0981 | cpfrc_00986 | CpI19_0985 | cauri_1092 | ----- | jk1200 | ----- | ----- |
| **ciuA** | Cp1002_0981 | CpC231_0982 | cpfrc_00987 | CpI19_0986 | cauri_2325 | DIP0582 | jk1296 | ckrop_0465 | cu0910 |
| **ciuB** | Cp1002_0982 | CpC231_0983 | cpfrc_00988 | CpI19_0987 | cauri_0361 | DIP0583 | jk1295 | ckrop_0415 | cu0399 |
| **ciuC** | Cp1002_0983 | CpC231_0984 | cpfrc_00989 | CpI19_0988 | cauri_0361 | DIP0584 | jk1294 | ckrop_1965 | cu0399 |
| **ciuD** | Cp1002_0984 | CpC231_0985 | cpfrc_00990 | CpI19_0989 | cauri_0721 | DIP0585 | jk1293 | ckrop_0466 | cu0335 |
| **ciuE** | Cp1002_0985 | CpC231_0986 | cpfrc_00991 | CpI19_0990 | ----- | DIP0586 | ----- | ----- | ----- |
|  | Cp1002_0986 | CpC231_0987 | cpfrc_00992 | CpI19_0991 | ----- | DIP0587 | ----- | ----- | ----- |
|  | Cp1002_0987 | CpC231_0988 | ----- | CpI19_0992 | ----- | DIP0230 | jk0343 | ----- | ----- |
|  | Cp1002_0988 | CpC231_0989 | cpfrc_00995 | CpI19_0993 | ----- | DIP0278 | ----- | ----- | ----- |
|  | Cp1002_0989 | CpC231_0990 | cpfrc_00997 | CpI19_0994 | ----- | DIP0278 | ----- | ----- | ----- |
|  | Cp1002_0990 | CpC231_0991 | ----- | CpI19_0995 | ----- | ----- | ----- | ----- | cu0012 |
|  | Cp1002_0991 | ----- | ----- | CpI19_0996 | ----- | ----- | ----- | ----- | ----- |
|  | Cp1002_0992 | CpC231_0992 | ----- | CpI19_0997 | cauri_1308 | DIP1201 | ----- | ----- | ----- |
| **PAI5 genes** |  |  |  |  |  |  |  |  |  |
|  | Cp1002_1445 | CpC231_1444 | ----- | CpI19_1451 | ----- | ----- | ----- | ----- | ----- |
|  | Cp1002_1446 | CpC231_1445 | cpfrc_01449 | CpI19_1452 | ----- | ----- | ----- | ----- | ----- |
|  | Cp1002_1447 | CpC231_1446 | cpfrc_01450 | CpI19_1453 | ----- | ----- | ----- | ----- | ----- |
|  | Cp1002_1448 | CpC231_1447 | cpfrc_01451 | CpI19_1454 | ----- | ----- | ----- | ----- | ----- |
|  | Cp1002_1449 | CpC231_1448 | cpfrc_01453 | CpI19_1455 | ----- | ----- | ----- | ----- | ----- |
|  | Cp1002_1450 | CpC231_1449 | cpfrc_01454 | CpI19_1456 | ----- | ----- | ----- | ----- | ----- |
|  | Cp1002_1451 | CpC231_1450 | cpfrc_01455 | CpI19_1457 | ----- | ----- | ----- | ----- | ----- |
| **putative RNA polimerase factor sigma 70** | Cp1002_1452 | ----- | cpfrc_01456 | ----- | ----- | ----- | ----- | ----- | ----- |
|  | Cp1002_1453 | ----- | cpfrc_01457 | ----- | ----- | ----- | ----- | ----- | ----- |
|  | Cp1002_1454 | CpC231_1453 | cpfrc_01458 | CpI19_1460 | ----- | ----- | ----- | ----- | ----- |
| **putative chromosome segregation ATPase** | Cp1002_1455 | ----- | cpfrc_01460 | ----- | ----- | ----- | ----- | ----- | ----- |
|  | Cp1002_1456 | CpC231_1455 | cpfrc_01461 | CpI19_1462 | ----- | ----- | ----- | ----- | ----- |
|  | Cp1002_1457 | CpC231_1456 | cpfrc_01462 | CpI19_1463 | cauri_1312 | DIP1895 | jk0526 | ----- | cu1796 |
| **Putative type III restriction-modification system** | Cp1002_1458 | ----- | cpfrc_01464 | ----- | ----- | ----- | jk1240 | ----- | ----- |
|  | Cp1002_1459 | CpC231_1458 | cpfrc_01466 | CpI19_1465 | ----- | ----- | jk1249 | ----- | ----- |
|  | Cp1002_1460 | CpC231_1459 | cpfrc_01467 | CpI19_1466 | ----- | ----- | jk1248 | ----- | ----- |
|  | Cp1002_1461 | CpC231_1461 | cpfrc_01469 | CpI19_1468 | ----- | DIP2314 | jk1254 |  | cu1265 |
|  | Cp1002_1462 | CpC231_1464 | cpfrc_01472 | CpI19_1471 | ----- | ----- | ----- | ----- | ----- |
|  | Cp1002_1463 | CpC231_1465 | ----- | CpI19_1472 | ----- | ----- | ----- | ----- | ----- |
|  | Cp1002_1464 | CpC231_1466 | cpfrc_01473 | CpI19_1473 | cauri_2127 | DIP0448 | jk0399 | ckrop_0057 | cu1587 |
|  | Cp1002_1465 | CpC231_1467 | cpfrc_01474 | CpI19_1474 | ----- | DIP0449 | ----- | ckrop_0056 | ----- |
| **htaC** | Cp1002_1466 | CpC231_1468 | cpfrc_01476 | CpI19_1475 | cauri_1722 | DIP1519 | jk0319 | ----- | ----- |
|  | Cp1002_1467 | CpC231_1469 | cpfrc_01477 | CpI19_1476 | ----- | DIP1520 | ----- | ----- | ----- |
| **pfoS** | Cp1002_1468 | CpC231_1471 | cpfrc_01478 | CpI19_1478 | cauri_1467 | ----- | ----- | ckrop_0280 | ----- |
| **guaB3** | Cp1002_1469 | CpC231_1472 | cpfrc_01479 | CpI19_1479 | cauri_0481 | DIP0580 | jk1723 | ckrop_0196 | cu0396 |
|  | Cp1002_1470 | ----- | ----- | ----- | ----- | ----- | ----- | ----- | ----- |
|  | Cp1002_1471 | CpC231_1473 | cpfrc_01480 | CpI19_1480 | cauri_0155 | DIP1660 | jk0589 | ----- | ----- |
|  | Cp1002_1472 | CpC231_1474 | cpfrc_01481 | CpI19_1481 | ----- | DIP1661 | jk0463 | ----- | ----- |
| **PAI6 genes** |  |  |  |  |  |  |  |  |  |
|  | Cp1002_1552 | CpC231_1554 | cpfrc_01561 | CpI19_1559 | cauri_0212 | DIP1743 | jk1711 | ckrop_1336 | cu0248 |
|  | Cp1002_1553 | CpC231_1555 | cpfrc_01562 | CpI19_1560 | ----- | ----- | ----- | ----- | ----- |
| **pipA1** | Cp1002_1554 | CpC231_1556 | cpfrc_01563 | CpI19_1561 | cauri_2086 | DIP1744 | ----- | ckrop_0537 | ----- |
|  | Cp1002_1555 | CpC231_1557 | cpfrc_01564 | CpI19_1562 | cauri_1662 | DIP1745 | jk0755 | ckrop_0752 | cu1200 |
|  | Cp1002_1556 | CpC231_1558 | cpfrc_01565 | CpI19_1563 | ----- | ----- | jk0156 | ckrop_0159 | cu0176 |
|  | Cp1002_1557 | CpC231_1559 | ----- | CpI19_1564 | ----- | ----- | ----- | ----- | ----- |
|  | Cp1002_1558 | CpC231_1560 | ----- | CpI19_1565 | ----- | ----- | ----- | ----- | ----- |
|  | Cp1002_1559 | CpC231_1561 | ----- | CpI19_1566 | ----- | ----- | ----- | ----- | ----- |
|  | Cp1002_1560 | CpC231_1552 | cpfrc_01567 | CpI19_1567 | cauri_0213 | DIP0325 | ----- | ckrop_1085 | ----- |
|  | Cp1002_1561 | CpC231_1551 | cpfrc_01568 | CpI19_1556 | cauri_1826 | DIP0324 | ----- | ckrop_1084 | ----- |
| **mfsD1** | Cp1002_1562 | CpC231_1564 | cpfrc_01569 | CpI19_1569 | ----- | DIP0323 | ----- | ckrop_1083 | ----- |
|  | Cp1002_1563 | CpC231_1565 | cpfrc_01570 | CpI19_1570 | cauri_1829 | DIP0322 | jk1593 | ckrop_1336 | cu0248 |
|  | Cp1002_1564 | CpC231_1566 | cpfrc_01571 | CpI19_1571 | cauri_1829 | DIP0321 | jk0654 | ckrop_0092 | cu0410 |
|  | Cp1002_1565 | CpC231_1567 | cpfrc_01572 | CpI19_1572 | ----- | ----- | ----- | ----- | ----- |
| **PAI7 genes** |  |  |  |  |  |  |  |  |  |
|  | Cp1002_1909 | CpC231_1903 | cpfrc_01915 | CpI19_1924 | cauri_2339 | DIP2133 | jk0177 | ----- | cu1817 |
|  | Cp1002_1910 | CpC231_1904 | cpfrc_01916 | CpI19_1925 | cauri_2341 | DIP2134 | jk1672 | ckrop_1544 | cu0443 |
| **arsR** | Cp1002_1911 | CpC231_1905 | cpfrc_01917 | CpI19_1926 | ----- | ----- | ----- | ----- | cu0047 |
| **phuC** | Cp1002_1912 | CpC231_1906 | cpfrc_01918 | CpI19_1927 | cauri_0721 | DIP1059 | jk1293 | ckrop_0466 | cu0400 |
| **fecD** | Cp1002_1913 | CpC231_1907 | cpfrc_01919 | CpI19_1928 | cauri_1724 | DIP1084 | jk0317 | ckrop_0415 | cu0399 |
| **fepC2** | Cp1002_1914 | CpC231_1908 | cpfrc_01920 | CpI19_1929 | ----- | DIP1086 | ----- | ckrop_0414 | cu0398 |
| **ureD** | Cp1002_1915 | CpC231_1909 | cpfrc_01921 | CpI19_1930 | ----- | ----- | ----- | ckrop_0803 | cu1777 |
| **ureG** | Cp1002_1916 | CpC231_1910 | cpfrc_01922 | CpI19_1931 | ----- | DIP0671 | ----- | ckrop_0804 | cu1776 |
| **ureF** | Cp1002_1917 | CpC231_1911 | cpfrc_01923 | CpI19_1932 | ----- | ----- | ----- | ckrop_0805 | cu1775 |
| **ureE** | Cp1002_1918 | CpC231_1912 | cpfrc_01924 | CpI19_1933 | ----- | ----- | ----- | ----- | cu1774 |
| **ureC** | Cp1002_1919 | CpC231_1913 | cpfrc_01925 | CpI19_1934 | ----- | ----- | ----- | ckrop_0806 | cu1773 |
| **ureB** | Cp1002_1920 | CpC231_1914 | cpfrc_01926 | CpI19_1935 | ----- | ----- | ----- | ckrop_0806 | cu1772 |
| **ureA** | Cp1002_1921 | CpC231_1915 | cpfrc_01927 | CpI19_1936 | ----- | ----- | ----- | ckrop_0808 | cu1771 |
|  | Cp1002_1922 | CpC231_1916 | cpfrc_01928 | CpI19_1937 | ----- | ----- | ----- | ----- | cu1790 |
|  | Cp1002_1923 | CpC231_1917 | ----- | CpI19_1938 | ----- | ----- | ----- | ----- | ----- |
| **alaT** | Cp1002_1924 | CpC231_1918 | cpfrc_01929 | CpI19_1939 | cauri_2342 | DIP2136 | jk0175 | ckrop_0271 | cu1835 |
|  | Cp1002_1925 | CpC231_1919 | cpfrc_01930 | CpI19_1940 | cauri_2344 | DIP2137 | ----- | ckrop_0270 | cu1769 |
| **lysS1** | Cp1002_1926 | CpC231_1920 | cpfrc_01931 | CpI19_1941 | cauri_2233 | DIP2138 | jk1200 | ckrop_1644 | cu1689 |
|  | Cp1002_1927 | CpC231_1921 | cpfrc_01932 | CpI19_1942 | ----- | DIP2139 | ----- | ----- | ----- |
|  | Cp1002_1928 | CpC231_1922 | cpfrc_01933 | CpI19_1943 | ----- | DIP2140 | ----- | ----- | ----- |
|  | Cp1002_1929 | CpC231_1923 | ----- | CpI19_1944 | cauri_2381 | ----- | ----- | ----- | ----- |
| **udg** | Cp1002_1930 | ----- | cpfrc_01934 | CpI19_1945 | cauri_2345 | DIP2141 | jk0173 | ckrop_0269 | cu1836 |
| **dcd** | Cp1002_1931 | CpC231_1925 | cpfrc_01935 | CpI19_1946 | ----- | DIP2142 | jk0174 | ckrop_0268 | cu1837 |

^a^ *Corynebacterium pseudotuberculosis* 1002; ^b^*Corynebacterium pseudotuberculosis* C231; ^c^ *Corynebacterium pseudotuberculosis* FRC41; ^d^ *Corynebacterium pseudotuberculosis* I19; ^e^ *Corynebacterium auricomucosum*; ^f^ *Corynebacterium diphtheriae*; ^g^ *Corynebacterium jeikeium*; ^h^ *Corynebacterium kroppenstedtti*; ^i^ *Corynebacterium urealyticum*.
